# Formation of disinfection by-products and fungal contamination data in public swimming pools: A case study in Gonabad, Iran

**DOI:** 10.1016/j.dib.2018.12.017

**Published:** 2018-12-12

**Authors:** Abbasali Karimi, Majid Radfard, Ali Naghizadeh, Hamed Biglari, Vida Alvani, Mokhtar Mahdavi, Alireza Mohammadzadeh

**Affiliations:** a Health Services Management, School of Public Health, Social Determinants of Health Research Center, Yasuj University of Medical Sciences, Yasuj, Iran; b Research Center for Health Sciences, Institute of Health, Department of Environmental Health, School of Health, Shiraz University of Medical Sciences, Shiraz, Iran; c Medical Toxicology and Drug Abuse Research Center (MTDRC), Birjand University of Medical Sciences (BUMS), Birjand, Iran; d Department of Environmental Health Engineering, School of Public Health, Social Development & Health Promotion Research Center, Gonabad University of Medical Sciences, Gonabad, Iran; e Department of Environmental Engineering, school of engineering and technology, Murdoch University, Western Australia, Australia; f Social Determinates of Health Research Center, Saveh University of Medical Sciences, Saveh, Iran; g Department of Microbiology, School of Medicine, Gonabad University of Medical Sciences, Gonabad, Iran

**Keywords:** Swimming pools, Disinfection, Fungi contamination, DBPs, HAAs, THMs, HANs

## Abstract

Existence of fungi and disinfection by-products (DBPs) in public swimming pools water are dangerous since it can seriously affect on health of swimmers. This data study aimed to determine the fungi contamination and DBPs concentration including trihalomethanes (THMs), haloacetic acids (HAAs), halamines and cyanogen halides and haloacetonitriles (HANs) of swimming pools (chlorine based) in Gonabad County, Iran. So, the fungal load and DBPs concentration were investigated in two swimming pools in the middle of spring of 2017 by collecting a number of 9 water samples and 9 samples of lateral facilities of each pool by membrane filtration technique and sterile carpet. The DBPs concentrations were measured by gas chromatograph technique. The results showed that the pools were contaminated with *Dermatophyte* (*trichophyton mentagrophytes* and *epidermophyton flucosomes*), *yeasts*, and more with opportunistic *saprophytic fungi*. 24.8%, 22.7%, 16.9%, and 11.4% *saprophytic fungi* were separated from pool side, locker room, pool water, and shower positions, respectively. 7.4% and 3.2% of yeast fungi as well as 0.23% and 0.2% of *dentofacies* of causative agents of *tinea* were separated from the pools water and showers as well as locker room and shower positions, respectively. According to the data, halamines and cyanogen halides had the highest concentrations, followed by HAAs, THMs and HANs respectively. Among the halamines and cyanogen halides, HAAs, THMs and HANs, trichloramine acid was the most dominant species, followed by trichloroacetic acid and dichloramine, respectively.

**Specifications table**

**Table:** 

Subject area	Environmental health sciences
More specific subject area	Environmental microbiology
Type of data	Tables
How data was acquired	The fungal contaminations and DBPs in two indoor swimming pools were investigated in the middle of spring of 2017 by collecting a number of 9 samples of water and 9 samples of lateral facilities of each pool by membrane filtration technique and sterile carpet. The final diagnosis of genus and species was carried out using slide culture and special media techniques. The DBPs concentration was determine by GC technique according to Standard Methods for the Examination of Water and Wastewater. The results were analyzed using descriptive statistics by excel software (v. 2017).
Data format	Raw, analyzed
Experimental factors	The collected samples were transferred into the laboratory of in less than 2 hours, then samples were passed through micro polar filters with 0.45 μm pores
Experimental features	The sample collection, fungal identification and DBPs analyses of selected swimming pools were done according to the standards method that presented in related valid references.
Data source location	Gonabad, Khorasan Razavi, Iran
Data accessibility	Data are included in this article

## Value of the data

• DBPs are the result of a reaction between the water disinfection materials and natural organic matters in the water. The concentrations of DBPs have been associated with negative health effects such as cancer and adverse reproductive outcomes.
• The data of this study could serve as a guide for future epidemiologic investigations exploring the potential relationships between DBPs in pools water and their adverse health effects.
• The data of this study showed that the total organic carbon content, chlorine quantity, bromide ion concentration and variations on THMs, HAAs and HANs formation potential in Gonabad public pools water that could use by environmental health researchers.
• The data confirmed that swimmers might result in greater DBPs absorption than simply drinking the tap water and need to be considered by health care institute.
• Drinking or contact with contaminated water containing a variety of pathogens is a major pathway of transferring diseases to humans and other organisms. In terms of contact with contaminated water, swimming pools are one of the most commonly infected environments.
• Fungal diseases are one of the most notable and most common diseases caused by the use of swimming pools. Therefore, continuous monitoring of swimming pool water is very important. The data of this study is in line with the mentioned goal.

## 1 Data

Access to safe water is vital for human life [1], [2]. Water disinfection is necessary for the protection of human health, which considerably diminishes mortality rates and the prevalence of fatal diseases [3]. The concentrations of some important physicochemical parameters in water samples of swimming pools are shown in Table 1. The average of temperature (°C), free chlorine (mg/l), total organic carbon (TOC) (mg/l) and pH in pools were 27.8, 1.29, 5.67 and 7.38, respectively. The maximum TOC concentrating observed about 9.1 mg/l. The types of fungal infections in two swimming pools environment are shown in Table 2. The fungi separated from the pools included *dermatophyte (trichophyton mentagrophytes* and *epidermophyton flocosum*), yeasts, and opportunistic saprophyte 0.4%, 7.93%, and 91.1%, respectively. As it can be seen in Table 2, a total of 1247 fungal colonies were separated from two indoor swimming pools in Gonabad of which 1137, 99, and 6 cases were mildew fungi, yeast fungi, and *dermatophytic* fungi colonies, respectively. In general, the major isolated *dermatophytic* fungi included *trichophyton mentagrophytes* and *epidermophyton flucosum*. The results showed that pool 1 with 57 yeasts has the highest yeasts and with 605 frequencies had the more mildew (Table 2). The obtained results indicated that the water in the examined pools did not have any dermatological contamination; however, there was a mold and yeast fungus in their water. The results also showed that pool 1 was contaminated with 82 and 18 and pool 2 with 56 and 24 colonies per liter of moldy mildew and yeast fungi.

**Table 1:** Concentrations of some important physicochemical parameters in water samples of swimming pools.

**Parameters**	**Pool one**	**Pool Two**
Temperature (°C)	27.3 ± 0.7 (24.2–28.6)	28.3 ± 0.5 (25.2–28.9)
Free chlorine (mg/L)	1.09 ± 0.70 (0.6–2.7)	1.49 ± 0.60 (0.3–2.3)
TOC (mg/L)	15.22 ± 1.62 (9.6–18.4)	26.12 ± 2.13 (12.8–29.1)
pH	7.13 ± 0.24 (7.4–7.8)	7.63 ± 0.14 (6.9–8.1)

**Table 2:** The types of fungal infections in two swimming pools environment.

**Pools**	***Trichophyton Mentagrophytes***	***Epidermophyton flocosum***	***Yeast***	***Mold***	**Total**
One	3 (0.4)	4 (0.1)	57 (9.5)	605 (90.0)	669 (100)
Two	2 (0.5)	2 (0.4)	42 (11.1)	532 (88.0)	578 (100)
Total	5 (0.40)	6 (0.48)	99 (7.93)	1137 (91.1)	1247 (100)

* Numbers in brackets represent percentages

The level of contamination in the locker room was different in the way that pool number 1 had the highest fungal contamination. Two *dermatophyte* colonies *were isolated* from locker room of pool 1 and no *dermatophytic* colonies were isolated from pool 2. From the shower room of the first pool, a case of *dermatophytic* colonies, and from shower room 2, two cases of *dermatophytic* colonies were isolated. The major moldy mildew saprophytic fungal were separated from *Aspergillus*, *Penicillium*, *Cladosporium, Alternaria, Fusarium, Mucor, Rhizopus* and yeast fungal of *Candida.Sp*, *Rhodotorula species*. According to the results of the study, *dermatophyte* fungal contaminations were more in dressing room and the margin of showers in indoor swimming pools of Gonabad compared to the other parts of the pools. In the study on the fungi found in the water of the pools, no *dermatophytic* fungi were isolated from the pool water, and the highest percentage of fungi present belonged to saprophytic fungi (1247 fungal colonies). Also, in the case of yeasts, the most and lowest frequencies were allocated to pools 1 and 2, respectively, while the highest and lowest frequencies of saprophytic fungi were included in pools 2 and 1, respectively. More than 90% of *saprophytic fungi* were *Aspergillus*, *Phenicillium*, and *Cladosporium*.

The most commonly classes of DBPs concentration in water environment of swimming pools are given in Table 3. The maximum concentration of tribromomethane, bromodichloromethane, dibromochloromethane and trichloromethane in THMs class were determined 110, 160, 90 and 211.2 µg/l, respectively.

**Table 3:** Disinfection by-products concentration in water environment of swimming pools in µg/l.

The maximum concentration of bromodichloroacetic acid, bromochloroacetic acid, dichloroacetic acid, chloroacetic acid, dibromoacetic acid, bromoacetic acid, dibromochloroacetic acid, trichloroacetic acid and tribromoacetic acid in HAAs class were obtained 21.71, 331.8, 492.34, 26.6, 12.6, 7, 28.1, 440.35 and 17 µg/l, respectively.

The maximum concentration of dichloramine, cyanogen chloride, monochloramine, trichloramine and cyanogen bromide in halamines and cyanogen halide class were measured 280.49, 19.37, 200.58, 750 and 12 µg/l, respectively.

The maximum concentration of trichloroacetonitrile, bromochloroacetonitrile, chloroacetonitrile, bromoacetonitrile, dichloroacetonitrile and dibromoacetonitrile in HANs class were observed 1.01, 5.21, 1.81, 1.2, 45 and 3.40 µg/l, respectively. Table 3 provides a more complete summary regarding the range occurrence of DBPs in water samples of swimming pools.

## 2 Experimental design, materials and methods

### 2.1 Sampling

**Fig. 1:**
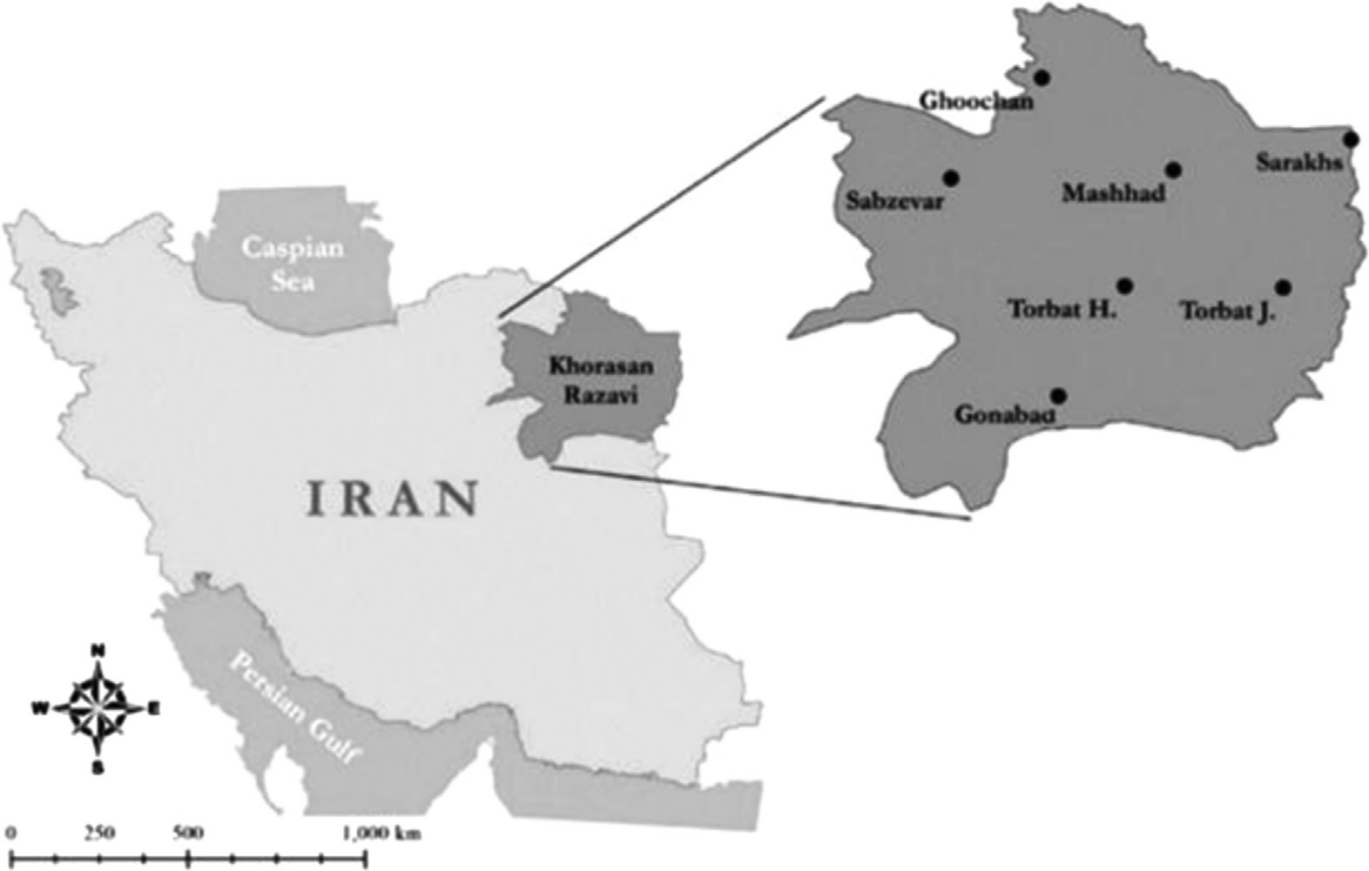
Gonabad geographical location.

In order to perform this data study, a number of 9 water samples and 9 samples of lateral facilities of two indoor swimming pools collected from Gonabad County, Fig. 1. Sampling from facilities prepared using membrane filtration technique and sterile carpet. Due to the limited availability of indoor pools and usage traffic, sampling was performed in mid-spring of 2017 when swimming pools had the most frequencies of visitors. Water samples were collected from the depth of 30 cm from the water surface and a 40 cm distance from the edge of the pool. Water and lateral facilities of each pool was sampled in a way that 9 samples were collected from each pool so that each sample contained 500 mL of water of the pools at the depth of 0.5, 30 cm and in 1.5 m longitudinal distances in a specific hour (between 12 and 14 o׳clock) in sterile bottles [4], [5]. The samples were transferred into the laboratory within 2 hours.

### 2.2 Fungi identification

The water samples were passed through micro polar filters with 0.45 μm pores. Then, millipol filters were transferred into saborud dextrose agar medium with sabouraud dextrose Agar + chloramphenicol + cycloheximide without sabouraud dextrose agar + chloramphenicol and Brain Heart Infusion Agar under sterile conditions and the cultures were kept at a temperature of 27 to 30 °C for three weeks and were examined for growth of fungal colonies daily [6]. The lateral facilities of pools including locker room, footbath and pool shower (and if there are a saunas and Jacuzzis) were sampled using sterile carpets with the sizes of 6 × 4 cm. For this purpose, 9 samples were taken from the walls and floor of related places in each pool and samples were immediately transferred to the laboratory. Then, the carpets were shaken on the mentioned culture media under sterile conditions so that the fungal elements in their warp and woof were transferred to culture media and the culture media were periodically examined. Then, various species of fungi were identified based on the colony characteristics in the environment and their microscopic structure which is done through cutting and side culture methods [7], [8]. To measure the DBPs levels, swimming pool samples were collected in 250 ml bottles. The water samples that collected for DBPs determine all kept in a dark glass bottles at the temperature of 4 °C until further assessment. Before sampling, approximately 2 mg of sodium thiosulfate was added to the bottles in order to neutralize residual chlorine [9].

### 2.3 Determination of DBPs

Free residual chloride was measured on the site using the colorimetric method by a portable colorimeter device. Then, water temperature and pH were analyzed on the site using a thermometer and portable pH meter, respectively. A TOC analyzer was applied to examine the TOC concentration in the water samples [10]. Before sampling, for determine HAAs and HANs compounds about 6 mg of ammonium chloride was added to the samples as the dechlorination agent for HAA determination [11], [12]. The potassium phosphate monobasic (KH_2_PO_4_) and 1% sodium phosphate monobasic (Na_2_HPO_4_), was used as a buffer to keep the pH to 4-5 and prevent HANs degradation. The water samples for DBPs determination were analyzed using a gas chromatograph based on the US EPA methods that was explained in Bahmani and Ghahramani and others studies [13], [14], [15].
