# Supplementary material for: Formation of disinfection by-products and fungal contamination data in public swimming pools: A case study in Gonabad, Iran
Source: Data Brief. 2018 Dec 12;22:326–31. doi: 10.1016/j.dib.2018.12.017 (PMC6307501; doi:10.1016/j.dib.2018.12.017)
Supplement: Supplementary file 2 — Supplementary material [file mmc2.pdf]

شماره : 123/97/ص/ات/ف/3325
تاریخ : 1397/8/15
پیوست :

بسمه تعالی
دانشگاه علوم پزشکی و خدمات بهداشتی درمانی گناباد
معاونت تحقیقات و فن آوری

Dear editor(s) Journal of Data in Brief,

We should informing to you that the paper “Formation of disinfection by-products and fungal contaminations data in public swimming pool water environment” written from the data of accepted research that has been supported by Gonabad University of Medical.

Kind Regards

دکتر میترا صالحی
سرپرست کمیته تحقیقات دانشجویی

استان خراسان رضوی-گناباد- معاونت تحقیقات و فن آوری دانشگاه علوم پزشکی و خدمات بهداشتی درمانی گناباد - کد پستی ۹۶۹۱۷۹۳۷۱۸-صندوق پستی ۳۹۷
سایت الکترونیکی www.gmu.ac.ir
تلفن ۳۰۱ داخلی ۱۴-۰۵۱-۵۷۲۲۳۰۲۸ نمابر ۰۵۱-۵۷۲۲۳۸۱۴
